# Growth Hormone and Insulin-Like Growth Factor Action in Reproductive Tissues

**DOI:** 10.3389/fendo.2019.00777

**Published:** 2019-11-12

**Authors:** Emina Ipsa, Vinicius F. Cruzat, Jackob N. Kagize, John L. Yovich, Kevin N. Keane

**Affiliations:** ^1^School of Pharmacy and Biomedical Science, Curtin University, Perth, WA, Australia; ^2^Faculty of Health, Torrens University Australia, Melbourne, VIC, Australia; ^3^PIVET Medical Centre, Leederville, WA, Australia

**Keywords:** estrogen, testosterone, granulosa cells, theca cells, Sertoli cells, Leydig cells, signaling

## Abstract

The role of growth hormone (GH) in human fertility is widely debated with some studies demonstrating improvements in oocyte yield, enhanced embryo quality, and in some cases increased live births with concomitant decreases in miscarriage rates. However, the basic biological mechanisms leading to these clinical differences are not well-understood. GH and the closely-related insulin-like growth factor (IGF) promote body growth and development via action on key metabolic organs including the liver, skeletal muscle, and bone. In addition, their expression and that of their complementary receptors have also been detected in various reproductive tissues including the oocyte, granulosa, and testicular cells. Therefore, the GH/IGF axis may directly regulate female and male gamete development, their quality, and ultimately competence for implantation. The ability of GH and IGF to modulate key signal transduction pathways such as the MAP kinase/ERK, Jak/STAT, and the PI3K/Akt pathway along with the subsequent effects on cell division and steroidogenesis indicates that these growth factors are centrally located to alter cell fate during proliferation and survival. In this review, we will explore the function of GH and IGF in regulating normal ovarian and testicular physiology, while also investigating the effects on cell signal transduction pathways with subsequent changes in cell proliferation and steroidogenesis. The aim is to clarify the role of GH in human fertility from a molecular and biochemical point of view.

## Introduction

Growth hormone (GH) is a 191 amino acid protein, which binds readily to the growth hormone receptor (GHR) and in some species the prolactin receptor (1, 2). The GHR is a member of the cytokine receptor superfamily (3) and although the majority of human GHR has been detected in the liver, it has also been found to be abundantly expressed in all cellular components of the human ovary and testes (4, 5). GH was demonstrated to have both direct and indirect effects on ovarian and testicular function, with direct effects mediated by the explicit GH-GHR interactions, while indirect effects likely to be mediated through the local production of secondary factors, particularly Insulin-like growth factor (IGF) (6), a protein that is typically produced by the liver in response to GH stimulation (1, 7, 8). Both GH and IGFs form part of the somatropic axis, which is markedly active at onset of puberty, and responsible for whole body growth and development (9). At puberty, animals also become sexually mature, and it is clear that the somatropic axis is connected to the establishment of reproductive function, but the precise mechanisms are still not fully understood (9, 10). While timing of puberty is genetically controlled (11), it is likely that the development of the body to a specific weight and/or size through the anabolic actions of GH and IGFs is at least partly responsible for onset of puberty (10, 12). This system is highly conserved from an evolutionary perspective, and has been observed across various animals including mammals and fish (10).

GH has been shown to have multiple specific effects in female and male reproductive physiology, such as promotion of steroidogenesis, enhancement of gonadotropin sensitivity as well as significant stimulatory effects on spermatogenesis and follicular development, which ultimately aligns with the initiation of puberty (4, 13, 14). This means that the GH-IGF system is likely to have profound effects on the major reproductive constituents of the ovary including granulosa cells, theca cells, and oocytes and in the testes including spermatids, Sertoli, and Leydig cells. Most of the biological understanding of the action of this system has been derived from animal studies, as access to developing human follicles from oophorectomy and testicular biopsy is limited. However, the specific biochemical interactions are under-researched. Nonetheless, we report here on the current knowledge regarding the biological and biochemical actions of both GH and the IGF system in female and male reproductive function, citing animal and where possibly, human studies. We will explore the effects of these proteins on follicular dynamics including growth and progression, proliferative effects on reproductive cells, production of key sex steroids such as testosterone, estrogen (E2), and progesterone (P4), the regulation by gonadotropins, and finally the intracellular signaling that mediate these activities.

## GH, GHR, and Follicular Growth

**Figure 1:**
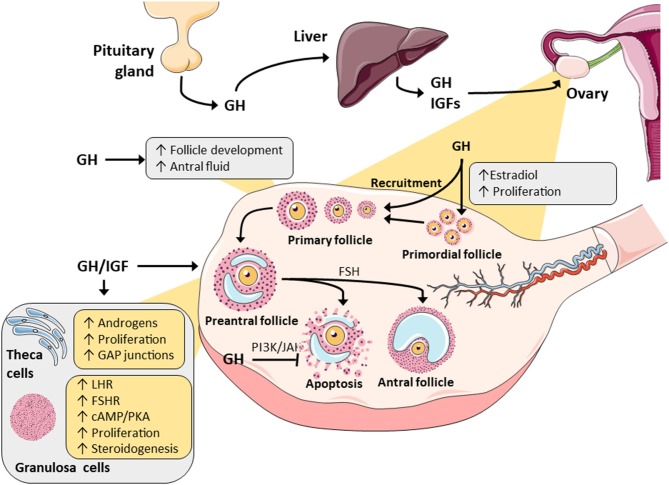
A summary of the major actions of GH and IGF in ovarian physiology. Both have been demonstrated to promote steroidogenesis in granulosa and theca cells through alterations in metabolizing enzymes. GH/IGF have also been reported to synergistically work with gonadotropins to alter steroidogenesis and this is possibly mediated by changes in the gonadotropic receptors. Finally, through intracellular signaling pathways (JAK/STAT and PI3/AK), GH and IGF may promote follicle selection and survival by decreasing follicular atresia.

GH has been reported by many studies to modify the growth of developing ovarian follicles (15–17). *In vitro* studies using caprine preantral follicles have demonstrated the stimulatory effect of GH on antral follicle development particularly during the initial antral phase (15). GH exposure over 18 days increased the diameter of caprine preantral follicles, and using *in vitro* maturation protocols, led to the generation of healthy oocyte-cumulus complexes, production of more metaphase II oocytes, and better fertilization ability (15). The same investigators showed that GH exposure over a similar period functioned synergistically with Follicle Stimulating Hormone (FSH) in supporting canine follicular growth, increasing the follicular diameter, promoting viability, and it was suggested that this was due to GH-induced production of antral follicle fluid and consequential antrum formation (Figure 1) (16). This response was largely observed in a separate study in secondary bovine follicles exposed to GH for 32 days, where the follicle diameter, antrum formation and E2 release were all increased (17).

The expression status of GHR mRNA at different follicle developmental stages was investigated in the goat, and high expression was found in oocyte, stromal, cumulus and mural granulosa cells of both small and large antral follicles (18). Interestingly, GHR was not detected in preantral follicles, and this may imply that any effect in the earliest follicular stages is mediated indirectly, possibly through the local GH-induced production of IGF, but in later, more mature follicles, they may respond directly to GH stimulation via the expression of the GHR. This observation was supported by another study where an elevated number of primordial and atretic follicles were found in GHR knock-out mice. They also showed a decreased number of primary, secondary, antral, and healthy growing follicles indicating failed follicular progression possibly due to the inability to upregulate sufficient GHR as follicles develop (19). Importantly, follicle progression was corrected with IGF-1 treatment (19), but this IGF-mediated effect was not observed in all GHR knock-out murine studies (20). Other investigations using knock-out animal models have provided further evidence to indicate that GH influenced reproduction, but was not completely essential for generating offspring. For example, while the absence of functional GHR was reported to cause an increase in systemic GH levels, a decrease in circulating IGF-1 level (but still present), and a delay in puberty onset with a reduced number of ovarian follicles, these animals could still reproduce, but with a smaller litter size (21–24). Several studies have confirmed that GHR knock-out resulted in a delay in puberty onset, and this echoes the delayed puberty that is observed in human disorders such as Laron dwarfism where GHR is dysfunctional (25, 26).

Taken together, it is reasonable to assume that the GHR influences fertility given its effect on puberty and that GH supplementation can restore fertility in humans with GH-deficiency (27). Furthermore, since the GHR was expressed on all cellular components of female adult follicles, it stands to reason that they contain the necessary cellular machinery for mediating direct actions (4). Moreover, membrane bound GHR was also reported to be expressed on the human oocytes, which suggested that GH may act directly on the oocyte itself, as well as indirectly via granulosa cells (28). However, GHR expression was not evident in fetal oocytes, perhaps indicating that it becomes active later in development, although this could be an artifact related to fetal termination prior to ovarian tissue extraction (4). Nonetheless, either directly or possibly indirectly through IGF-1, GH was demonstrated to play major role in primordial follicular growth and progression in various animal models and consequently it may regulate the recruitment of primordial follicles into the growing, gonadotropin-sensitive pool (16, 18). This is possibly one reason as to why beneficial effects are observed with GH supplementation during IVF treatment (29, 30).

## GH and Ovarian Cell Proliferation, Differentiation, and Gonadotropin Response

There is evidence to suggest that GH and/or IGF act in synergy with gonadotropins, FSH and luteinising hormone (LH), in reproductive tissue to promote granulosa and theca cell expansion, along with granulosa cell differentiation to luteal cells (Figure 1). In rat ovaries (31), GH treatment in the presence of FSH enhanced granulosa cell differentiation, but there was no change in cell proliferation. Conversely, in mouse follicles, addition of GH enhanced both granulosa and theca cell proliferation (Figure 1) (32). However, it has remained unclear whether GH induces cell expansion directly through GHRs expressed on these cells, or indirectly via stimulation of secondary growth factors produced by granulosa cells, such as IGF, which would then directly act on theca cells (32, 33). The GH-induced response in theca cell proliferation was confirmed in ovine *ex vivo* models, where the high concentrations of GH caused excessive growth of theca cells, such that they depleted nutritional elements in the medium (34). This effect was further corroborated in an *in vitro* study, where high doses of GH were found to be harmful to rat preantral follicle survival, possibly due to excessive theca and stromal cell proliferation and subsequent nutrient depletion (35). Due to these proliferative effects and expansion of follicular cells, the addition of GH to alginate-based growth media containing bovine secondary follicles produced higher levels of E2 synthesis and secretion (17). It was also noted that this increase in E2 production could contribute to the preservation of follicular architecture and function, and lead to better follicular development (17).

**Figure 2:**
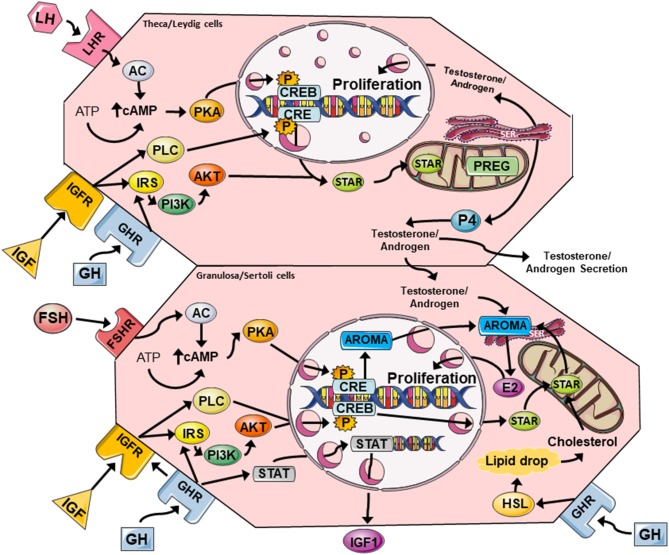
A summary of the major GH and IGF signaling networks in female (theca/granulosa cell) and male (Leydig/Sertoli cell) reproductive physiology. Both GH and IGF can activate PLC/PKC and PI3K/Akt pathways that cross-talk with FSHR and LHR signaling via cAMP/PKA to promote steroidogenesis and cell proliferation. Steroidogenic events are mediated by CREB-dependent expression of aromatase (granulosa cells), and StAR expression in all cell types. StAR allows cholesterol to enter the mitochondria where it can be converted to PREG, and then subsequently to testosterone/androgens, estrogens, and progesterone. Estrogens and testosterone enhance cell proliferation via autocrine mechanisms, while GH can induce local IGF expression in granulosa and Sertoli cells via JAK/STAT signaling. LHR, luteinising hormone receptor; FSHR, follicle stimulating hormone receptor; AC, adenylate cyclase; cAMP cyclic AMP; PKA, protein kinase A; CRE, cAMP response element; CREB, cAMP response element binding protein; PLC, phospholipase C, IRS, insulin receptor substrate; PI3K/Akt, phosphoinositide 3-kinase/protein kinase B; StAR, steroidogenic acute regulatory protein; PREG, pregnenolone; SER, smooth endoplasmic reticulum; P4, progesterone; E2, estradiol; STAT, signal transducer and activator of transcription; AROMA, aromatase, HSL, hormone-sensitive lipase.

Interaction of FSH and LH with their complementary gonadotropin receptors (i.e., FSHR and LHR) induces downstream signaling that is critical for steroidogenesis, proliferation, and differentiation, and both signal through the cyclic adenosine monophosphate/protein kinase A (cAMP/PKA) pathway to enhance production of E2 and P4 (Figure 2). FSHR and LHR are both G-protein coupled receptors (GPCRs) that transmit the intracellular cascade via adenylate cyclase activity, cAMP accumulation, with subsequent activation of PKA, which then phosphorylates the transcription factor CREB (cAMP response element binding protein). CREB binds cAMP response elements (CRE) in genomic DNA causing the transcription of various genes including those encoding for steroidogenic enzymes (e.g., aromatase) and cholesterol transport, the precursor substrate for sex steroid synthesis (e.g., steroidogenic acute regulatory protein, StAR).

The GH-GHR interaction in granulosa cells can modulate FSH action and also induce the expression of LHR (31, 36). This downstream expression of LHR is a key marker of granulosa cell differentiation to luteal cells, and can also possibly be influenced by GH stimulation of IGF within the ovary, which acts in a paracrine manner when it augments granulosa cell expansion (33, 37). The reported effect of GH on FSHR and LHR expression *in vitro* (31) and *in vivo* (38) is not trivial. This indicated that GH may modify or potentiate the sensitivity of granulosa cells and/or theca cells to gonadotropin stimulation and subsequently regulate sex steroid synthesis and release in follicles, which then boosts cell growth as paracrine/autocrine steroidogenic factors (Figure 1) (39). The two cell theory explains that ovarian steroidogenesis is regulated by consequent and mutually dependent processes (40), where LH stimulates theca cells to produce androgens, which are converted to various estrogens by the aromatase enzyme expressed in granulosa cells under the induction by FSH (40). Prior to oocyte release, granulosa cells become luteinised by upregulating LHR expression, responding to human chorionic gonadotropin (hCG), and producing progesterone thus forming the corpus luteum in the secretory phase of the menstrual cycle.

GH was shown to promote androsterone and androgen synthesis in rat theca cells, and this response was independent of IGF production and cAMP accumulation (Figures 1, 2) (6). In rat granulosa cells, co-treatment with FSH and GH significantly enhanced LHR expression and increased P4 synthesis and secretion, but there was no change in E2 production or cell proliferation (31). Central to these effects in granulosa cells was a clear enhancement of FSH-induced accumulation of cAMP, which is a key mediator of steroidogenesis, hormone receptor formation and differentiation of granulosa cells into luteal cells (41). Interestingly, *in vivo*/*ex vivo* studies in women with decreased ovarian reserve revealed that GH supplementation as part of IVF treatment, increased the expression of LHR, FSHR, and GHR in human granulosa cells isolated after egg collection (28, 38). GH also acted on supporting the maturation process of luteinization by increasing LHR density and by reducing the expression of FSHR prior to ovulation (38). The cytosolic accumulation of cAMP and activation of PKA signaling can also be triggered via GHR- and IGFR (insulin-like growth factor receptor) cross-talk, and this is likely to influence gonadotropin responses (42). The convergence of gonadotropin response and these pathway on steroidogenesis in female and male reproductive tissues is discussed below.

## Ovarian GH-IGF Axis

Interaction of GH with GHR can activate canonical and non-canonical downstream signaling. In canonical signaling, pituitary GH stimulates liver cells to release IGF into the circulation through transcription factors activated by GH-GHR. The ligand-receptor interaction triggers recruitment and autophosphorylation of JAK2 (Janus kinase) at the cytoplasmic domain of the GHR, and the GHR/JAK2 complex subsequently phosphorylates STAT (signal transducer and activator of transcription) molecules (particularly STAT5a, 5b, 1, and 3), which translocate to the nucleus and modify gene transcription leading to significant effects on cell proliferation (43). STAT5b is of most importance and directly regulates the expression of IGF-1 (43, 44), and was demonstrated to be a mediator of GH-induced IGF-1 production in rat granulosa cells (Figure 2) (33, 45, 46). Non-canonical GH-GHR intracellular signaling is typically independent of JAK2, and involves recruitment of Src family non-receptor tyrosine kinases (45), stimulation of phospholipase Cγ (PLCγ) and via cytosolic calcium flux from organelles, activation of protein kinase C (PKC) (47). As explained later, these components can cross-talk with other pathways such as the MAPK/ERK1/2 (mitogen activated protein kinase/extracellular signal-regulated kinase 1/2) and PI3/Akt (phosphoinositide 3-kinase/protein kinase B) signaling cascades causing changes in gene expression and modifying cell metabolism and proliferation (47).

It is not obvious which hormone or system is more important, as ovarian function can be influenced by systemic GH and IGF, local GH, GH-induced local IGF, and/or GH-independent IGF (48). However, it is clear that the GH-IGF axis is a key growth factor system involved in folliculogenesis (49). GH was shown to increase IGF-1 mRNA expression in rat preantral follicles (50) and promoted IGF-1 secretion from ovine granulosa cells (51). Furthermore, addition of IGF binding protein-3 (IGFBP-3) antagonized the anti-apoptotic effects of GH, which suggested that exogenous GH promoted local IGF-1 production that prolonged follicular survival (51). Consequently, the interplay of the GH-IGF system in the ovary is complex, as it can be utilized in paracrine and autocrine processes by granulosa cells and does not necessarily require GH stimulation.

The systemic IGF system is made up of IGF-1 and IGF-2 (52), type 1 and type 2 IGF receptors (IGF-1R &−2R) and six IGF binding proteins (IGFBP 1-6) (53), that regulate IGF bioavailability (54). However, paracrine expression of these components is also important during ovine folliculogenesis, and the local level of IGF is enhanced by the decreased expression of IGF biding proteins (IGFBP-2,-3,-4,-5, and−6) in growing follicles, as they advance from potential atresia to follicle selection (55). Expression of IGF-2 was decreased in atretic follicles while IGF-2 R and IGFBP-5 was significantly increased in atretic follicles (55). These data indicated that reducing the local bioavailability of IGF leads to follicle demise simultaneously suggesting that IGF expression is key for follicle survival and possibly selection (54). The level of ovarian IGF is also related to the stage of folliculogeneiss, with low levels detected in theca cells derived from medium sized follicles and in oocyte from infants (56), while higher levels of IGF-2 expression were observed in granulosa cells isolated from large antral follicles (54). Therefore, it appears that there is a dynamic requirement for GH and IGF activity as follicles mature and grow.

Analogous to GH, IGF-1 stimulates proliferation and differentiation of granulosa cells and theca cells (57, 58). It does so by also potentiating FSH actions on granulosa cells, and it was demonstrated that the IGFR was absolutely required for FSH-mediated activation of the PI3K/Akt pathway which is a pro-survival cascade, and subsequent granulosa cell differentiation (59, 60). Zhao et al. found that the presence of IGF-1 stimulated cell proliferation in rat primordial follicles by measuring the increasing DNA content within the follicular cells (61). They also noted that cells cultured with IGF-1 exhibited better morphology due to the increased number of gap junctions between theca-granulosa cells and granulosa cell-oocyte (61). They detected 80% more cortical granules underneath the oocyte membrane with IGF-1 exposure, and hypothesized that it potentially accelerated the development of the preantral oocyte cytoplasm. In addition, the presence of FSH and IGF-1 improved preantral follicular growth due to the activation of the FSHR (61). The stimulatory effects of IGF-1 on follicular and cell survival have also been shown in caprine preantral follicles and oocytes (62, 63), in porcine granulosa cells (64) and in bovine antral follicles, oocytes and granulosa cells (65). An *in vitro* study performed by Magalhães-Padilha et al., demonstrated a higher growth rate of IGF-1 stimulated caprine preantral follicles and they stipulated that it was most likely due to cellular proliferation, as it was demonstrated that IGF-1 enhanced nuclear maturation of granulosa cells in preantral follicles (63).

Animal studies using genetic knock-outs also demonstrated a more direct role for IGFs over GH in reproduction. For example, female mice with IGF-1R knock-out were shown to be completely sterile, with no antral follicles and a 90% reduction in serum E2 levels (66). In fact, inactivation of either IGF-1 or IGF-R by knock-out is incompatible with life in the majority of the cases, and in the rest of the cases, it certainly causes infertility in both sexes with an infantile reproductive system (67). Moreover, IGF-1 knock-out mice exhibited significantly reduced expression of FSH receptors and consequently reduced aromatase expression and E2 secretion (68), indicating that IGF-signaling may regulate gonadotropin receptor expression.

The combination of FSH and presence of the IGFR leads to various intracellular signaling events such as cAMP production, which as outlined in turn activates PKA and CREB, along with activation of the MAPK/ERK1/2 and PI3K/Akt pathways (60, 69). These signaling mechanisms increase aromatase activity and LHR expression (54). To induce aromatase activity, FSH, and IGF-1 or−2 work in synergy and act on their respective receptors (FSHR and IGF-1R) (60). IGF-1 has specific and stimulatory effects on granulosa cells, and it was reported to increase the expression of steroidogenic enzymes CYP11A1, 3β-hroxysteroid dehydrogenase (3βHSD), CYP19A1, along with IGF-1R, and FSHR gene expression (70). It was noted that IGF-1 activated steroidogenic and apoptotic regulatory genes through activation of PI3K/Akt pathway in bovine granulosa cells (70, 71). Both IGF-1 and IGF-2 can stimulate the production of sex steroids involved in follicular development. IGF-1 together with LH enhanced granulosa cell P4 production and acted as regulator of E2 synthesis in luteal cells (71). Importantly, the IGF-1R is also critical for the increased expression of StAR under FSH stimulation, which is required for mitochondrial transport of cholesterol for the first step of sex steroid synthesis, pregnenolone production (60). Furthermore, it has been demonstrated that high concentrations of GH/IGF supress the activity of Anti-Müllerian hormone (AMH), which is exclusively secreted in gonadal tissues (72). AMH is one of the members of transforming growth factor beta (TGF-β) super-family of growth factors, and downregulates both development and the function of preantral and antral follicles in primates (73, 74). This action may partially explain the role of GH/IGF in regulating follicular development and selection. Taken together, these data indicated clearly that IGF plays a central role in regulating follicular development via granulosa cell proliferation, differentiation, steroid production, and by mediating the stimulatory activity of gonadotropins. These effects along with that of GH are summarized in Table 1.

**Table 1:** Summary of major findings from GH and IGF studies in ovarian and testicular physiology.

**Gender/Factor**	**References**	**Model/Tissue type/Cell type**	**Major effects mediated by factor**		
**FEMALE**					
**Growth hormone**					
	Araújo et al. (17)	Bovine follicles	↑ Antrum formation	↑ Estadiol concentration	–
	Sirotkin and Makarevich (33)	Bovine granulosa cells	↑ IGF-I secretion	↑ IGFBP-3 secretion	↓ Presence of regulatory PKA subunit
	Serafim et al. (16)	Canine follicles	↑ Antrum formation	↑ Estradiol secretion	↑ Follicular diameter
	Magalhaes et al. (15)	Caprine follicles	↑ Antrum formation	↑ M2 oocyte yield	↑ Nuclear maturation
	Martins et al. (18)	Caprine ovaries	↑ Development of preantral follicle	GHR mRNA detected in antral follicles	GHR mRNA not present in preantral follicles
	Weall et al. (28)	Human COC oocytes	↑ Oocyte mitochondrial function	↑ oocyte quality	GHR detected on human oocyte
	Regan et al. (38)	Human granulosa cells	↑ Density of FSHR, BMPR1B, LHR, and GHR	–	–
	Kobayashi et al. (32)	Murine preantral follicles	↑ Granulosa cell proliferation	↑ Theca cell proliferation	–
	Arunakumari et al. (34)	Ovine preantral follicles	↑ Development of preantral follicle	↑ Nuclear maturation of the oocyte	–
	Khalid et al. (51)	Ovine granulosa cells	↑ IGF-I secretion	↑ Estradiol secretion	↑ porgesterone secretion
	Apa et al. (6)	Rat theca cells	↑ Androstendione sythesis	↑ Androgen production	–
	Jia et al. (31)	Rat granulosa cells	↑ FSH-stimulated LH receptor count	↑ FSH-stimulated progesteron secretion	↑ FSH-stimulated 20 alpha-hydroxy-4-pregnen-3-one secretion
	Eisenhauer et al. (50)	Rat preovulatory follicles	↓ Follicle cell apoptosis	↑ GH-induced IGF mRNA	–
	Zhao et al. (35)	Rat preantral follicles	↑ Growth of preantral follicle	↑ Morphology quality of preantral follicle	–
			↑ Presence of catalytic PKA subunit	↓ Progesterone secretion	↓ Apoptosis incidence
**Insulin-like growth factor**					
	Walters et al. (65)	Bovine antral follicles	↑ Follicular size	↑ Estradiol secretion	↑ oocyte health
	Mani et al. (70)	Bovine granulosa cells	↑ Proliferation	↑ Estradiol secretion	↑ CYP11A1, HSD3B1, CYP19A1, BAX, IGF1R and FSHR expression
	Zhou and Zhang (62)	Caprine preantral follicles	↑ Proliferation	↑ Preantral follicle survival rate	-
	Magalhaes-Padilha et al. (63)	Caprine preantral follicles	↑ Percentage of normal follicles	↑ Rate of antrum formation	↑ Meiotic resumption rates
	Baumgarten et al. (60)	Human cumulus granulosa cells	↑ Proliferation	↑ Differentiation	PI3K/AKT mediated
	Zhou et al. (68)	Murine ovary	↑ Granulosa cell FSHR expression	–	–
	Hastie and Haresign (55)	Ovine ovary	↑ IGF-2 in large follicles	↓ IGF-II in atretic follicles	↑ IGFBP-5 in artretic follicles
	Campbell et al. (58)	Ovine & bovine granulosa cells	↑ Cell proliferation	↑ Oestradiol secretion	–
	Guthrie et al. (64)	Porcine granulosa cells culture	↓ Spontaneous apoptosis	–	–
	Zhao et al. (61)	Rat preantral follicles	↑ Follicular diameter	↑ Folicular morphology	↑ Cortical granules
**MALE**					
**Growth hormone**					
	Sjogren et al. (75)	*In vivo* canine treatment	↓ Testicular and prostatic weight	–	–
	Matsushima et al. (76)	*In vivo* GH/thyroxine deficient mice	↑ Seminiferous tubule cell count	↑ Sperm count	↓ FSH levels
	Piotrowska et al. (77)	Murine testes	↑ Testicular size	↑ Testicular aging	↓ LHR & AR
	Ovesen et al. (78)	*In vivo* humans	↑ Serum/seminal IGF-I and serum IGFBP-3	↑ Sperm motility	↑ IGF
	Arsenijevic et al. (79)	*In vivo* rat testicles	↓ Testicular and seminal vesicle size	↓ Spermatogenesis	-
	Kanzaki and Morris (80)	Rat Leydig cells	↑ Androgen production	↑ StAR activity	↑ 3β-HSD mRNA expression
**Insulin-like growth factor**					
	Dance et al. (81)	Bovine Sertoli cell culture	↑ Cell proliferation	–	–
	Bingol-Kologlu et al. (82)	Murine germ cells	↑ Haploid cell number	–	–
	Saez et al. (83)	Porcine Leydig and Sertoli cell lines	↑ Stimulatory effect of FSG on cAMP production in Sertoli cells	↑ Pregnenalone to testosterone conversion	↑ Plasminogen activator

## Role of GH in Testes

Expression of GH has been detected across various systems in the human including neural, immune, respiratory, and reproductive tissues (84, 85). There are two clinically important versions of GH, including the normal GH form (GH-N) secreted from the pituitary gland, and the variant GH form (GH-V), first detected in the placenta (84). While both isoforms have been detected in the human testes, as well as in the testes of other mammals, GH-V was shown to be predominantly expressed form in human testes (86, 87). The level of testicular GH was found to be significantly less than that observed in the pituitary, and thus testicular GH is expected to act locally and not systemically (84). GH receptors and GH binding proteins were also observed to be abundantly expressed throughout the male reproductive system, in Leydig cells, Sertoli cells, seminal vesicles, epididymis, vas deferens, and prostate (5). However, the majority of the GH-induced effects on seminiferous tubules and testicular growth have been found to be indirect, and mainly accomplished via IGF generation and action (88, 89). Importantly, IGFR was reported to be expressed in the male reproductive tract including localization in early spermatids, secondary spermatids, Sertoli cells, and to a lesser extent Leydig cells (90, 91). Interestingly, men with distal chromosome 15 structural abnormalities are more likely to experience cryptorchidism, and this appears to involve the IGFR locus (92). Therefore, it is reasonable to expect that the GH-IGF axis could regulate aspects of male reproductive function and development.

GH is accepted to play an important role in sexual maturation in all mammalian species, and is an important contributor to the onset of puberty (14). Both in humans (93) and in experimental animals (94), pubertal delay was observed with GH deficiency (83). During male puberty, GH has roles in testicular development and differentiation, stimulation of germinal cell differentiation (79), influencing increased testicular diameter (8), and aiding in the development of the Wolffian ducts (89), all of which are underdeveloped in GH-knock-out mice (95). Furthermore, there is a positive correlation between serum IGF levels and testicular volume (95, 96), and administration of GH can significantly accelerate puberty if onset has been delayed (90, 97). In Laron dwarfism, due to the insensitivity to GH, and in GHR knock-out mice, appropriate testicular function develops later than in healthy males, but still occurred (88). This indicated that testicular development may be only partly GH-dependent, with the majority of stimulatory effects on testes mediated directly by IGF (88).

Moreover, gametogenesis is significantly influenced by GH. Ovesen et al. demonstrated an increase in sperm motility in GH-treated men and an increase in semen volume in oligospermic men treated with GH (78). In addition, it was found that GH supplementation caused an increase in germ cell number and an improvement in sperm morphology (82, 88). The potential mechanism by which GH may improve spermatogenesis is possibly through the stimulation of Leydig and Sertoli cell differentiation (14). Furthermore, GH was found to improve Leydig cells responsiveness to physiologic human chorionic gonadotropin (hCG), a key hormone regulating spermatogenesis (8).

When GH-deficient rats were treated with GH, it was demonstrated to have a protective effect on the count and motility of spermatids following treatment with cyclophosphamide (98) In addition, it prevented testicular atrophy and testosterone depletion after treatment with methotrexate (88, 99). Both agents are important chemotherapeutics used in cancer treatment, and thus GH supplementation may play a role in preservation of fertility with cancer therapy.

Male GH knock-out mice have a significantly lower cell number in seminiferous tubules, which with corresponding underdevelopment of sperm, decreases fertility (76). The effects of GH on testicular development are evident in its stimulatory action on Leydig cell maturation and proliferation (77, 80). GH promoted androgen production along with StAR and 3β-HSD expression in progenitor Leydig cells (80). It was suggested that this action was mediated by activation of STAT5-dependent steroidogenesis by GH and through stimulation of Leydig cell proliferation (14, 80), although other studies in animals failed to demonstrate any androgenic effect (88, 100). In the latter situation, it could be that GH-mediated enhancement of aromatase activity leads to an increase in testosterone to E2 conversion, reducing testosterone levels but increasing E2 (72, 88). However, administration of GH and subsequent GH-mediated effects were found to be dose dependent, as high concentrations given to GH-deprived canines caused atrophy of testes and accessory organs, thinning of prostatic epithelium and a reduction in LH and testosterone levels (75). In addition, overexpression of GH in mice led to early testicular aging characterized by lower expression of the androgen receptor (AR) and LHR (77). Conversely, GH deficit does not supress ongoing spermatogenesis, although GH treatment has the ability to restore inhibited spermatogenesis possibly indicating a potentiating function (101). These findings indicate that the response of male reproductive organs to GH is complex.

## Role of IGF in Testes

Interestingly, GH-induced IGF secretion by Sertoli cells was reported to increase the number of LH receptors in Leydig cells, meaning that IGF could increase testicular androgen production (8, 83, 90), and both IGF-1 and IGF-2 were shown to enhance testosterone production (90). IGF also has proliferative actions on Sertoli cells in the same autocrine manner (90). As stated before, GH-induced effects on seminiferous tubules are mainly accomplished through IGF action (88, 89). IGFR expression has been shown in porcine Leydig and Sertoli cells (83). The majority of the IGF-1 effects on Leydig cells was found by examining knock-out mice. IGF-1 knock-out mice exhibited significant stunting in the development of vas deferens, seminal vesicles and prostate, along with developmental delay of Leydig cells, which were fewer than normal (67, 102). In addition, testosterone levels were reduced by 82%, and LH-stimulated testosterone production was decreased (67). However, capacitated sperm from these mice were able to fertilize oocytes (67). Interestingly, IGF regulates Leydig cell differentiation and stimulates hCG-dependent cAMP synthesis in order to stimulate steroidogenesis (90, 103). hCG was also shown to upregulate the expression of IGFR in rat Leydig cells suggesting cross-talk between both pathways (90, 103). Furthermore, IGF was demonstrated to increase responsiveness of porcine Leydig cells to physiological hCG concentrations and to pharmacological steroidogenesis activators (83).

In cattle, IGF induced proliferation of Sertoli cells by 18% and was crucial for maintaining the Sertoli population (81). This stimulatory effect was enhanced significantly when IGF functioned in unison with FSH, echoing the response observed in female granulosa cells (81). It has been reported that IGF promoted thymidine inclusion in DNA of Sertoli cells and to have role as a mitogenic stimulator in immature Sertoli cells (8). Furthermore, it can regulate glucose and lactate metabolism in Sertoli cells, which are crucial metabolites for germ cell health (95). It also stimulates plasminogen activator production in Sertoli cells (8, 83, 95), which is secreted by Sertoli cells and plays an important role in germ cell development, formation, and migration (104). These effects in testicular biology, along with that of GH are summarized in Table 1.

## GH-IGF Signaling: Convergence With Gonadotropin Signaling

While male and female reproductive systems are clearly different, the response to gonadotropins including cell proliferation and sex steroid synthesis/release is largely biochemically similar (Figure 2). It is likely that any effects GH and/or IGF have on male and female reproductive physiology is mediated through changes in these pathways, and potentiation of subsequent steroidogenesis, the products of which have their own effects on cell proliferation and survival. In this final section, we describe the intersecting biochemical points of gonadotropin and GH/IGF signaling.

Stimulation of the FSHR by FSH leads to activation of the cAMP/PKA pathway and subsequent CREB-mediated transcription of various genes. This process can upregulate the expression of steroidogenic genes such as those encoding aromatase and StAR, along with the LHR gene (105, 106). The aromatase gene is directly regulated by CREB (107), and this enzyme converts androgens (e.g., testosterone) to estrogens, while StAR mediates the transport of cholesterol substrate to the mitochondria for synthesis of testosterone, E2 and P4 in steroidogenic cells (Figure 2). However, the activation of FSHR and LHR GPCRs will trigger other key cell signaling events, that can also impact on steroidogenesis. One central pathway is the PI3K/Akt cascade, which is a well-known regulator of cell metabolism, proliferation and survival (108), and can be directly stimulated by FSHR following direct interaction with 14-3-3τ adaptor proteins (109). The PI3K/Akt pathway is also stimulated by LH, with its activity heightened in the presence of FSH (110, 111).

Akt is a multifunctional signaling hub that can regulate cell metabolism, proliferation, and death (36, 95, 112). FSH-mediated activation of Akt is essential for the expression of 3β-HSD, α-inhibin, CYP19, LHR (113), and there is accumulating evidence to indicate that FSHR-mediated aromatase expression requires both cAMP/PKA and PI3K/Akt activation (59, 60, 114). Interestingly, recent research in human and rodent granulosa cells has shown that intact IGF-1R signaling was also required for FSHR-mediated phosphorylation of Akt (66, 113). It is beginning to emerge that FSHR action requires obligatory PI3K/Akt signaling, and achieves this by supporting IGF-IGFR stimulation of Akt. This is evident from studies showing that FSH could not promote CYP19, LHR, and StAR expression in the presence of an IGF inhibitor (113). GH and IGF intracellular signaling are both connected to FSHR and LHR signaling via the regulation of the insulin signaling pathway which incorporates the PI3K/Akt cascade. IGF and proinsulin share homology such that both of their respective receptors, IGFR and the insulin receptor (IR), will bind to the alternate growth factors albeit with reduced affinity (3). Interaction of IGF with the IR leads to the recruitment and phosphorylation of insulin receptor substrate 1 or 2 (IRS1 or 2) with subsequent activation of PI3K then Akt (3). IRS1/2 are possibly the key intermediates between FSH and IGF-PI3K/Akt activation, as it has recently been proposed that in Sertoli cells, PKA stimulation by FSH leads to enhanced activity of protein phosphatase 1 (PP1), which can promote IRS signaling by dephosphorylating inhibitory serine/threonine residues (95). However, further studies are required, particularly in male reproductive organs, to confirm if this mechanism exists.

The vast majority of GH actions are mediated through the JAK-STAT signaling events which has multiple complex roles, such as regulating cell proliferation and oocyte maturation (Figure 1) (115, 116) and significant downstream crosstalk with the other pathways (42, 117). GH is likely to be involved in the above process simply by its ability to upregulate local IGF production through classical JAK/STAT signaling, but JAK2 can also directly cross phosphorylate IRS1/2 adding another link to the GH-GHR cascade (33, 118, 119). GH-GHR initiation of STAT5b can promote the expression of local IGF which then acts in an autocrine manner to stimulate PI3K/Akt signaling and enhances FSH-FSHR activities (Figure 2). Importantly, outside of JAK-STAT and PIK3/Akt, the GH-GHR, IGF-IGFR, IGF-IR interactions can stimulate several other different intracellular signaling cascades notably PLC/PKC and MAPK/ERK1/2 pathways (120).

A key aspect of GH and IGF stimulation of the PLC/PKC pathway, is the ability to promote CREB-mediated transcription. For GH-GHR, non-canonical intracellular signaling, which is independent of JAK2 involves recruitment and activation of Src family non-receptor tyrosine kinases (45). Src family molecules such as Shc and Lyn, interact with the cytoplasmic domain of the GHR and activate phospholipase Cγ (PLCγ) which then proceeds to hydrolyse phospholipids to form inositol-1,4,5 triphosphate (IP3) and diacylglycerol (DAG) (47). These components go on to increase cytosolic calcium flux from organelles and activate PKC, respectively. PKC activity is critical because it can also trigger CREB-mediated gene transcription directly, and thus StAR and/or aromatase expression for example (121). It was shown that stimulation of PKC led to enhanced StAR expression and progesterone secretion in Leydig and granulosa cells (Figure 2) (121, 122). For IGF, the IGFR can directly activate PLCγ also leading to the above signaling cascade. Interestingly, the PKC pathway can also be activated by FSHR via formation of IP3 and DAG and this leads to the expansion of cumulus cells and meiotic maturation of oocytes (123), again neatly demonstrating crosstalk between gonadotropin GPCRs and GH/IGF signaling., The interconnection of these signaling systems at least partly explains the physiological effects observed *in vitro*/*ex vivo*.

One final convergence point of these related signaling events, is the initiation of the p38 MAPK and MAPK/ERK1/2 signaling pathways, that causes changes in gene expression and can modify cell metabolism and proliferation (47). ERK1/2 functions to enhance mitogenic signals in cells and can be activated by elevated intracellular calcium (from PLC/PKC events), and indirectly via PKA, both of which as outlined are stimulated by GH and IGF activity (106, 124). In granulosa cells, p38 MAPK plays a role in generating pro-apoptotic signals (124). In Sertoli cells, PKA stimulates MAPK/ERK1/2 and this leads to FSH-induced cell proliferation (125). However, the contribution of the MAPK/ERK1/2 signaling pathway to steroidogenesis is less clear. For example, MAPK activation is important for FSH-mediated StAR and progesterone synthesis, while blocking this cascade increases aromatase activity and E2 production (106, 126). However, in another study, IGF-mediated stimulation of progesterone synthesis and secretion in human ovarian cells is dependent on MAPK/ERK1/2 and p38 MAPK signaling (127). Both MAPK/ERK1/2 and the pro-survival PI3K/Akt pathways are stimulated by IGF-I, IGF-2, and activated IGF-1R in cumulus granulosa cells (60). Thus, this interplay and individual participation of MAPK signaling in steroidogenesis remains unclear.

## Future GH-IGF Mechanisms to Explore and Concluding Remarks

Since steroid hormones are not stored in large quantities in steroidogenic tissues, there is constant demand for cholesterol, the main precursor of steroidogenesis (128). Consequently, steroidogenic cells have numerous, small lipid droplets that contain cholesteryl esters that release free cholesterol upon stimulation by hormones. Two enzymes are important for liberating cholesterol, hormone sensitive lipase (HSL) and adipose triglyceride lipase (ATGL). HSL was found to be expressed in internal and external theca cells as well as granulosa cells of preantral follicles (129). ATGL was detected in granulosa and Leydig cells (128, 130). Both enzymes are responsible for 90% of lipolysis, and the activity of HSL is mediated through phosphorylation at various serine residues. PKA which is active in response to FSH, stimulates HSL activity via phosphorylation at Ser-563, Ser-650, and Ser-660 (42, 130). It was also observed that GH increases HSL mRNA and protein expression in mice, illustrating the direct regulatory role of GH in lipolysis (42, 131, 132). Conversely, it has been reported that GH may indirectly enhance ATGL expression *in vivo* through an unknown mechanism, but the effects of ATGL in steroidogenic cells in the ovary and testes requires further research (133). In catabolic conditions, the PLC, PKC, and MAPK/ERK signaling cascades play an important role in activating HSL and releasing lipids for energy production and as outlined, both GH and IGF can activate these pathways (46). Interestingly, it has been shown that ERK can directly phosphorylate HSL at Ser-600 increasing the enzyme activity in adipocyte cell lines (42, 134). GH and IGF have the ability to alter the direction of lipid metabolism via regulation of these enzymes and this may have important implications for lipid homeostasis in steroidogenic cells and tissues, especially those derived from the reproductive system where GH is used regularly as an adjuvant for fertility treatment. However, little research has explicitly explored this area in reproduction.

The downstream signaling from FSH-FSHR interactions is clearly central to the life and death balance observed in granulosa cells and developing follicles, and seems to have parallels in male reproductive cells. Some reports have indicated that altered FSH-signaling and/or over expression of FSHR can actually promote apoptosis in unselected follicles, which may potentially happen via excessive accumulation of cAMP or activation of p38 MAPK pathways (106, 135). It could be the case that the pro-survival signals mediated by GH and/or IGF, prevent pro-apoptotic events, and thus have a largely beneficial effect on male and female reproductive cell proliferation. The downstream FSHR and LHR signaling cascades are very diverse, but it is clear that there is significant cross-talk with pathways associated with GH and IGF signaling. GH is regularly used as an adjuvant in fertility treatment, and studies in animal and *ex vivo* human models demonstrate that GH and IGF regulate steroidogenesis, cell proliferation, and follicular development. While this area of research has undoubtedly progressed, it is still not completely clear which biochemical mechanisms are involved.

## Author Contributions

The concept of this review was designed by JY and KK. The initial manuscript draft was undertaken by EI and KK. VC, JK, and JY contributed substantially to manuscript revision. Figure 1 was designed by EI, KK, and VC. All artwork produced by VC.

### Conflict of Interest

The authors declare that the research was conducted in the absence of any commercial or financial relationships that could be construed as a potential conflict of interest.
